# Mechanism of Fumonisin Self-Resistance: *Fusarium verticillioides* Contains Four Fumonisin B_1_-Insensitive-Ceramide Synthases

**DOI:** 10.3390/toxins16060235

**Published:** 2024-05-22

**Authors:** Tamara Krska, Krisztian Twaruschek, Gerlinde Wiesenberger, Franz Berthiller, Gerhard Adam

**Affiliations:** 1 Institute of Microbial Genetics, Department of Applied Genetics and Cell Biology, BOKU University, Konrad-Lorenz-Strasse 24, 3430 Tulln, Austria; tamara.krska@boku.ac.at (T.K.); krisztian.twaruschek@boku.ac.at (K.T.); gerlinde.wiesenberger@boku.ac.at (G.W.); 2 Austrian Competence Centre for Feed and Food Quality, Safety and Innovation FFoQSI GmbH, Konrad-Lorenz-Strasse 20, 3430 Tulln, Austria; 3 Institute of Bioanalytics and Agro-Metabolomics, Department of Agrobiotechnology (IFA-Tulln), BOKU University, Konrad Lorenz Strasse 20, 3430 Tulln, Austria; franz.berthiller@boku.ac.at

**Keywords:** fumonisin, self-resistance, ceramide synthase, gene disruption, heterologous expression, target insensitivity

## Abstract

*Fusarium verticillioides* produces fumonisins, which are mycotoxins inhibiting sphingolipid biosynthesis in humans, animals, and other eukaryotes. Fumonisins are presumed virulence factors of plant pathogens, but may also play a role in interactions between competing fungi. We observed higher resistance to added fumonisin B_1_ (FB_1_) in fumonisin-producing *Fusarium verticillioides* than in nonproducing *F. graminearum*, and likewise between isolates of *Aspergillus* and *Alternaria* differing in production of sphinganine-analog toxins. It has been reported that in *F. verticillioides*, ceramide synthase encoded in the fumonisin biosynthetic gene cluster is responsible for self-resistance. We reinvestigated the role of *FUM17* and *FUM18* by generating a double mutant strain in a *fum1* background. Nearly unchanged resistance to added FB_1_ was observed compared to the parental *fum1* strain. A recently developed fumonisin-sensitive baker’s yeast strain allowed for the testing of candidate ceramide synthases by heterologous expression. The overexpression of the yeast *LAC1* gene, but not *LAG1*, increased fumonisin resistance. High-level resistance was conferred by *FUM18*, but not by *FUM17*. Likewise, strong resistance to FB_1_ was caused by overexpression of the presumed *F. verticillioides* “housekeeping” ceramide synthases *CER1*, *CER2*, and *CER3*, located outside the fumonisin cluster, indicating that *F. verticillioides* possesses a redundant set of insensitive targets as a self-resistance mechanism.

## 1. Introduction

Sphingolipids are abundant in the membranes of eukaryotes but also exist in some prokaryotes [1]. In eukaryotes, they are involved in processes like membrane trafficking, cell signaling, apoptosis, and others. Furthermore, disturbances in sphingolipid metabolism have been implicated in a variety of human diseases [2]. The sphingolipid core structure consists of a long acyl chain amide, which is linked to a fatty acid by ceramide synthase [3]. The long chain base in animal ceramides is sphingosine, while in plants and fungi, sphingolipid biosynthesis starts by the condensation of the tri-hydroxylated long chain base phytosphingosine with an alpha-hydroxylated very long chain fatty acid [4]. Phosphosphingolipids have a polar headgroup linked to ceramide via a phosphodiester bond. Highly complex structures [5,6] exist in different organisms with different roles due to the attachment of inositol(-phosphates) and different sugar moieties. 

Fumonisins are the major group of “sphinganine analog mycotoxins” [7], alongside the AAL toxin produced by *Alternaria alternata* f.sp. *lycopersici*. Fumonisin B_1_ (FB_1_) in particular is known to efficiently inhibit ceramide synthase in plants [8,9] and animals [10] by competing with sphinganine and acyl-coenzyme A [11,12]. Disturbances of sphingolipid biosynthesis have many effects: FB_1_ is a potential human carcinogen (group 2B according to the International Agency for Research on Cancer), further implicated in esophageal cancer and neural tube defects in humans, and known to cause animal diseases such as equine leukoencephalomalacia, porcine pulmonary edema and cancer. Also, teratogenic, mutagenic, cytotoxic, nephrotoxic, neurotoxic, and immunotoxic effects have been described [13,14,15]. 

The main producers of different fumonisins are plant pathogenic fungi, such as different species of *Fusarium*, several species of black *Aspergilli* and also *Verticillium* and some *Alternaria* strains [16]. Yet, *Alternaria alternata* f.sp. *lycopersici* typically produces the structurally related AAL toxin (see [7] for review). The gene clusters for fumonisin biosynthesis in different fungi have been elucidated [7,17,18,19]. 

Whether fumonisin production is a virulence factor of plant pathogenic fungi is a controversial issue. Fumonisin-deficient *fum1* mutants of *F. verticillioides* were still able to cause Fusarium ear rot in maize [20]. An *F. verticillioides* strain from banana (now *F. musae*) containing a large deletion of the FUM cluster was not pathogenic to seedlings of maize. Yet, when the FUM cluster was added back by transformation and fumonisin biosynthesis was restored, it gained virulence [21]. Also, inactivation of *fum1* in several strains led to reduced stunting of seedlings, indicating that it is a virulence factor in seedlings at least in some sensitive maize cultivars. Maize can have highly variable resistance to FB_1_ in a seed germination assay [22]. For *F. proliferatum*, which causes rice spikelet rot disease, it was shown that the disruption of several genes leading to loss of fumonisin production caused reduced virulence [23]. Also, in *Verticillium dahliae* causing wilting disease in cotton, fumonisin-deficient knockout strains were less virulent [24]. In the case of *Alternaria alternata* f.sp. *lycopersici*, which causes stem canker on susceptible tomato cultivars, resistance to the AAL toxin leads to resistance against the fungal pathogen (host selective toxin) [25]. Tomatoes with a homozygous loss of function of *Asc1*, encoding a ceramide synthase, are susceptible to the toxin and to the fungus [26]. Similarly, in *Arabidopsis*, inactivation of one of three ceramide synthase genes in this species, *LOH2*, leads to toxin sensitivity and breakdown of non-host resistance against an AAL-producing *Alternaria alternata* [27]. 

*F. graminearum* and *F. verticillioides* can co-occur and compete in infected maize ears. In a recent study [28], no significant difference between wild-type and *fum1* mutants in disease severity or amount of fungal DNA in the inoculated maize line was found. Yet, it was demonstrated that wild-type *F. verticillioides* could suppress the growth of *F. graminearum* in a co-culture on autoclaved kernels more strongly than a fumonisin-nonproducing strain. The authors hypothesized that fumonisin production in seeds suppresses colonization by other fungi after the seeds have been shed and that the main function of fumonisins thereby is to increase saprophytic fitness. 

Data on fumonisin resistance or susceptibility in different fungi are scarce. It has been reported that FB_1_ in very high concentrations (200 µL of up to 40 mM—corresponding to mg amounts per well in the agar) produced large growth inhibition zones with isolates of *Botrytis cinerea* and (not AAL-toxin-producing) *A. alternata* from a South African collection, while *F. graminearum* showed much higher resistance [29]. Conversely, Dawidziuk et al. [30] reported that a *Fusarium graminearum* isolate from Poland showed strong growth retardation by fumonisin already at the low concentration of 3 mg/L FB_1_ mixed into the agar medium, while *F. oxysporum* and *F. proliferatum* isolates were unaffected by this concentration. 

In principle, very high concentrations of fumonisins and also AAL toxin can be produced in fungal cultures and some mechanism of self-resistance must exist in toxin-producing fungi. Recently it has been reported that in the case of *Fusarium verticillioides*, self-protection against FB_1_ is conferred by a FUM cluster-encoded ceramide synthase [31]. 

The aim of our study was to test whether *Fusarium*, *Aspergillus*, and *Alternaria* strains producing sphinganine-analog mycotoxins have higher levels of FB_1_ resistance than related non-producers. Testing by gene disruption revealed that the cluster-encoded ceramide synthases of *F. verticillioides* are unexpectedly NOT necessary for high-level resistance. This result is explained by our finding that three presumed housekeeping ceramide synthases, when expressed in a sensitive yeast strain, are sufficient to confer high-level FB_1_ resistance.

## 2. Results

### 2.1. Sphinganine-Analog Producing Fungal Species Are More Resistant to Fumonisin B_1_ Than Non-Producers

**Figure 1:**
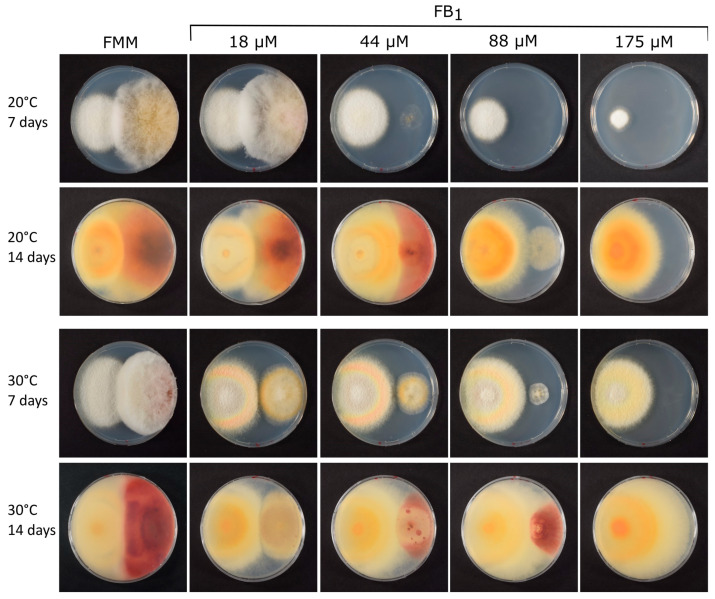
Growth of *F. verticillioides* and *F. graminearum* on FMM medium containing FB_1_ (crude extract) at different temperatures. Pictures were taken after the indicated incubation time (on day 7 from above and on day 14 taken from below for better visualization of the red *F. graminearum* pigment).

To investigate whether the production of fumonisins or the related AAL toxin is associated with increased toxin resistance, we compared the growth of various fungal strains (see Table 1) in the presence of FB_1_. First, we compared the growth of a well-studied *F. verticillioides* strain (FGSC 7600), which had been previously utilized for elucidation of the FUM cluster and for determination of the first genome sequence [32], with the growth of the likewise relevant fumonisin-nonproducer *F. graminearum* (strain PH-1, [33]) at different temperatures and different levels of fumonisins added to minimal medium. Since very high concentrations were needed for full inhibition, a crude concentrated extract containing fumonisins B_1_, B_2_, and B_3_ was used as previously described [34], which contained 3.18 g/L FB_1_. Without added toxin at 20 °C, *F. graminearum* (red pigmented, on the right half of the plates shown in Figure 1) grew more vigorously and covered a larger portion of the medium than *F. verticillioides*. At 30 °C, *F. verticillioides* grew better, and after two weeks, both strains covered about half of the plate. When increasing amounts of fumonisin were added to the medium, *F. graminearum* was increasingly inhibited, while *F. verticillioides* continued to grow. At the highest concentration tested (176 µM FB_1_), growth of *F. graminearum* was completely inhibited, while *F. verticillioides* showed only marginally reduced radial growth after 7 days at 30 °C (Figure 1). We conclude that the fumonisin-producing *F. verticillioides* has clearly higher resistance to fumonisin than *F. graminearum*.

**Figure 2:**
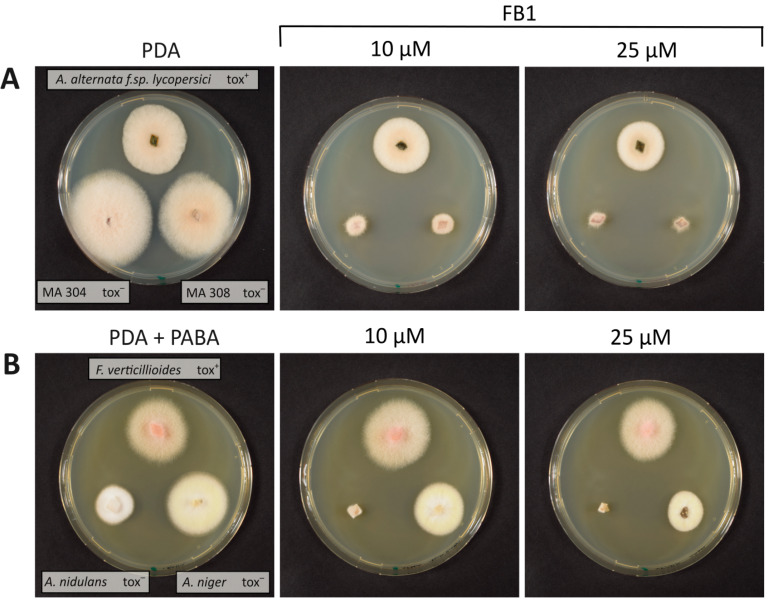
(**A**) Growth of *Alternaria* strains on FB_1_ containing PDA medium. Small agar blocks of the indicated *Alternaria* strains (AAL toxin producer (tox^+^) on top, the two nonproducers (tox^−^) below) were transferred to PDA plates containing the indicated amount of FB_1_. (**B**) Growth of *A. nidulans* (not fumonisin producing) and *A. niger* on PDA plates supplemented with PABA (p-aminobenzoic acid (PABA), 1.0 mg/L) and containing the indicated concentration of FB_1._

Next, we compared various *Alternaria* strains (Figure 2A) producing or not producing sphinganine-analog toxins. The *A. alternata* f.sp. *lycopersici* strain AS27-12 is a well-known producer of AAL toxin and related derivatives [35]. Its resistance level was compared to two *A. alternata* isolates from our local university collection (Austrian Center for Biological Resources (https://acbr-database.boku.ac.at/, accessed on 21 May 2024)). The strain MA 304 was originally isolated from apple in the USA, whereas MA 308 caused leaf spot in *Solanum tuberosum*. Both strains do not produce AAL toxin. Already, at the low concentration of 10 µM (about 7.2 mg/L), the growth of both nonproducing strains was strongly reduced to about 20% of the diameter, while the AAL-producing strain had 78% of its diameter on the no-toxin control. At 50 µM FB_1_, the AAL strain had an about 50% reduced diameter, while the two nonproducer strains were almost completely inhibited.

We also tested (Figure 2B) whether an *Aspergillus niger* wild-type strain, for which fumonisin production had been demonstrated (ATCC 11414, [36], see Table S1 therein), is more resistant than a wild-type *A. nidulans* strain (FGSC A4, [37]). *F. verticillioides* was added on top of the plates as a control (Figure 2B). At 10 µM FB_1_, the *A. nidulans* strains were already fully inhibited, while *A. niger* was only slightly inhibited (compared to no toxin 86% diameter at 10 µM and 66% at 50 µM). Seemingly, a mechanism of protection exists in sphinganine-analog toxin producers. We set out to test whether target insensitivity is involved.

### 2.2. Generation and Characterization of a fum17-18 Deletion Strain in a fum1 Background

**Figure 3:**
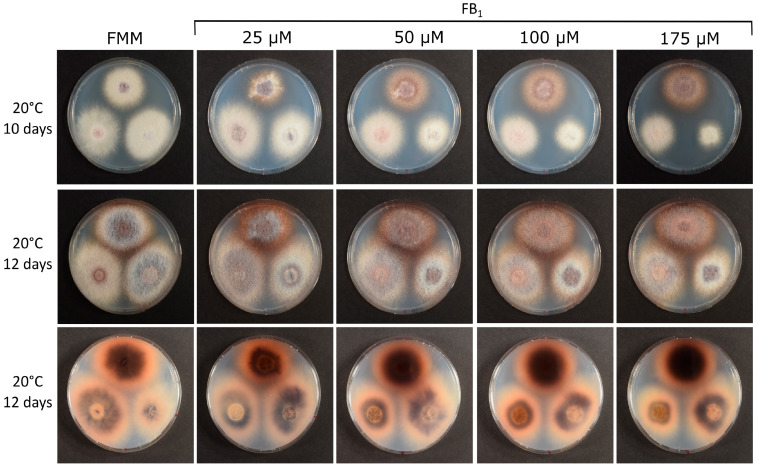
Growth of Δ*fum1* and two Δ*fum1* Δ*fum17-18 (*double mutants, KTFD1 and KTFD4) mutants on FB_1_-containing plates. The *fum17*-*fum18* (**bottom**) were inoculated onto FMM plates containing different concentrations of crude FB_1_ together with the parental *fum1* (**top**). Strains were grown for 12 days with pictures taken after 10 and 12 days. The bottom row shows the backside of the plates after 12 days (note, that plates are mirrored).

To be able to study the effects of added toxin undisturbed by endogenously synthesized fumonisin, we generated a *fum17*-*fum18* double mutant in the background of a *fum1*::*hygB* mutant. The previously described *fum1* mutant strain GfA2364, which is derived from the wild-type strain FGSC 7600 [20,38] by insertion of the *hygB* resistance gene into the *FUM1* PKS, was transformed with a construct that allows for simultaneous deletion of both genes using a *nptII* (G418) resistance cassette (see Section 4). Two transformants, designated KTFD1 and KTFD4, were obtained and used in the fumonisin resistance tests: their growth was compared to the growth of the parental *fum1* mutant strain GfA2364. As evident from Figure 3—even on the highest concentration tested—both, the wild-type and the knockout strains were still able to grow. For unknown reasons, stronger and earlier pigmentation occurred in the wild type. We conclude that the cluster-encoded ceramide synthase genes *FUM17* and *FUM18* are not necessary for high-level resistance to fumonisin B_1_.

### 2.3. Testing Fumonisin Resistance of Ceramide Synthase Genes by Heterologous Expression in Yeast

The finding that the *FUM17* and *FUM18* genes are not necessary for self-resistance against FB_1_ indicates possible redundancy. *F. verticillioides* has three additional predicted ceramide synthase genes, *CER1*, *CER2*, and *CER3* [31], encoded outside the FUM cluster. We have recently reported the construction of a fumonisin-sensitive *Saccharomyces cerevisiae* strain [34]. We transformed this strain with the plasmids described by Janevska et al. [31] for the expression of the *F. verticillioides* ceramide synthase cDNAs behind the constitutive *TEF1* promoter. As controls, the yeast ceramide synthases *LAG1* (“longevity assurance gene”, [39]) and its paralog (“longevity assurance cognate”) *LAC1* [40] were also overexpressed. Yeast transformants were spotted onto SC-URA plates supplemented with increasing amounts of FB_1_. Overexpression of *LAC1*, but not of *LAG1*, conferred low-level resistance at concentrations that were inhibitory for the empty vector controls. At higher concentrations, the yeast genes did not confer resistance, in contrast to *F. verticillioides FUM18*, *CER1*, *CER2*, and *CER3*.

**Figure 4:**
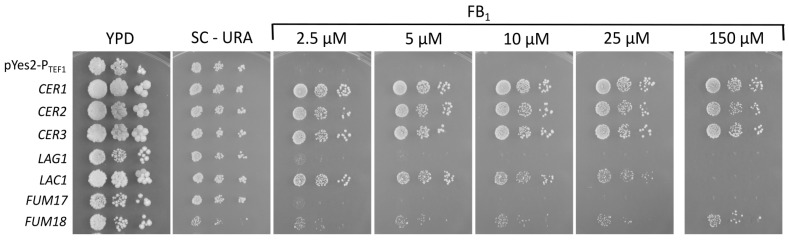
Growth of transformants of the FB_1_-sensitive *Saccharomyces cerevisiae* strain YTKT33 on URA-dropout SC agar media containing increasing concentrations of FB_1_. For the highest concentration, 75% pure FB_1_ was used. YTKT33 was transformed with the empty expression vector, pYes2-P_TEF1_ (negative control), or expression vectors containing: *F. verticillioides* ceramide synthase *CER1*, *CER2*, *CER3*, the two *S. cerevisiae* ceramide synthases *LAG1* and *LAC1*, and two putative ceramide synthase genes from the *F. verticillioides* fumonisin cluster, *FUM17* and *FUM18*.

As shown in Figure 4, the yeast host (containing functional endogenous *LAG1* and *LAC1* genes) transformed with the empty vector was already sensitive to 2.5 µM FB_1_. Overexpression of *LAC1* but not *LAG1* in the 2 µ multicopy plasmid behind the strong *TEF1* promoter conferred a low level of increased resistance. On the other hand, high-level resistance (highest concentration tested 150 µM) was conferred by the expression of *FUM18* but not *FUM17*, and equally well by all three *F. verticillioides* ceramide synthases, *CER1*, *CER2*, and *CER3*.

## 3. Discussion

The *FUM* cluster of *F. verticillioides* contains two genes, *FUM17* (FVEG_00327) and *FUM18* (FVEG_00328), which have sequence similarity to ceramide synthases. It has previously been reported [41] that both *FUM17* and *FUM18* expression were upregulated by FB_1_ addition to the medium. We used a *fum1* background to avoid contribution by differences in endogenous FB_1_ production to the overall effect. Both genes, which are located next to each other (with overlapping 3′ ends of the mRNAs), had been inactivated simultaneously by the insertion of a hygromycin resistance gene, which did not lead to a significant reduction of fumonisin production [12]. More recently, it was reported that “self-protection against the sphingolipid biosynthesis inhibitor fumonisin B_1_ is conferred by a FUM cluster-encoded ceramide synthase” [31]. Using an assay supposedly reflecting fungal biomass, which is based on the activation of resazurin (a dye that is converted into the fluorescent derivative resorufin by respiratory activity), more relative inhibition (about 120% compared to wild-type) in liquid culture was observed upon addition of FB_1_ [31]. No direct evidence for reduced growth of the knock-out strain on a solid medium was shown. 

Our results confirm that the *FUM18* gene is sufficient to confer fumonisin resistance when expressed in fumonisin-sensitive yeast. The double mutant *lag1 lac1* is lethal in most yeast strains. The *FUM17* plasmid did not complement the conditional yeast mutant [31] when doxycycline was added in order to switch off the expression of the integrated tetracycline-regulated promoter P_TET_-*LAG1* gene in the Δ*lac1* background. In agreement with this finding, we did not observe increased resistance compared to the empty vector in our sensitive yeast strain. It has been reported that *FUM17* is obviously non-functional in two other strains of the *F. fujikuroi* species complex [41], and more subtle mutations may also lead to the inactivity of the *F. verticillioides FUM17* gene product. While *FUM18* is sufficient to confer resistance in yeast, it is surprisingly not necessary for the high-level resistance to FB_1_ in *F. verticillioides*. The knockout mutants (*fum17-18* double mutant, similar to that described but with a different selection marker, *fum17-18*Δ::*HSVtk-nptII*) in the *fum1*::*hygB* background are hardly inhibited in growth on solid medium by added FB_1_ (Figure 3). The observed effect of inactivating the cluster ceramide synthases on FB_1_-mediated growth inhibition is minor, but unexpectedly, differences in the amount and timing of pigment formation were observed between independent *fum1 fum17-18* and *fum1* mutants. Since both strains are *fum1* mutants, this should not be due to an alteration of the metabolic flux from fumonisin into a different metabolite that is responsible for this phenotype. Further research would be necessary to elucidate which changes occur at the level of the transcriptome or metabolome. The result that *FUM18* is not necessary for fumonisin resistance can be explained by our finding that other ceramide synthases of *F. verticillioides* also confer high-level resistance in yeast. A surprising result is the high level of resistance conferred by *CER3* (FVEG_15375), as it was reported that this gene is not able to complement the yeast ceramide synthase’s loss of function [41]. Janevska et al. [41] reported that in the resazurin assay, overexpression of *CER1* (FVEG_06971) and *CER2* (FVEG_06971) showed a slightly reduced growth inhibition compared to the empty vector. In our strain, the same overexpression plasmids conferred high-level resistance (no evident inhibition at 175 µM FB_1_) and the transformants were growing at least as well as the *FUM18*-overexpressing strain (see Figure 4). 

The finding that *FUM18* is not necessary for self-resistance is in agreement with results with an *alt7* knockout strain in *A. alternata* producing AAL toxin. *ALT7* is a ceramide synthase gene located in the cluster for AAL toxin biosynthesis. The knockout of *ALT7* had no deleterious effect on the AAL toxin-producing pathogen, so the authors concluded that the gene does not act as a resistance/self-tolerance factor [42]. 

For the fungi that we tested, the hypothesis holds true that fumonisin producers are more resistant to FB_1_ than related non-producers. Several black *Aspergilli* can produce fumonisins B_2_, B_4,_ and B_6_, e.g., on grapes [43] or maize [44], although the levels are typically lower than in *Fusarium*. The non-producing model fungus *A. nidulans* turned out to be extremely sensitive to added FB_1_; growth was already fully inhibited by 10 µM FB_1_. The FUM cluster of *A. niger* does not contain a ceramide synthase [45], but it nevertheless showed higher resistance than *A. nidulans* and it potentially also has “housekeeping” ceramide synthase genes responsible for the higher FB_1_ resistance. In agreement with the reported much higher resistance in *F. graminearum* [29] and in contrast to Dawidziuk et al. [30], we found that the *F. graminearum* strain PH-1, lacking a *FUM18* ortholog, displayed quite high FB_1_ resistance (Figure 1). If the fungus–fungus competition hypothesis is meaningful, as obviously production of very high levels of fumonisins is needed in this scenario. 

Numerous cases exist (for review see [46]) where duplicated housekeeping genes containing sequence alterations encode insensitive target enzymes. These are often associated with toxin or antibiotic biosynthetic gene clusters, which allow for the elucidation of the mode of action of some compounds [47,48]. The presence of a (duplicated) putative self-resistance gene in a secondary metabolite biosynthetic cluster has been successfully used to identify new compounds with a desired mode of action, for instance, in the case of aspterric acid of *A. terreus* targeting branched-chain amino-acid biosynthesis [49]. Yet, the conclusion that such enzymes are also necessary for self-resistance may not be correct. Our results show that in the case of *F. verticillioides*, the fumonisin-cluster-encoded ceramide synthase *FUM18* is not necessary for self-resistance due to redundancy in self-resistance genes, as three other ceramide synthases, *CER1–3*, are additionally sufficient to confer fumonisin resistance. 

## 4. Materials and Methods

### 4.1. FB_1_-Sensitivity of Growth of Fusarium and Other Fungi

*F. verticillioides* (FGSC 7600) and *F. graminearum* (PH-1) were activated on Fusarium minimal medium (FMM; 1 g/L KH_2_PO_4_, 0.5 g/L MgSO_4_·7H_2_O, 0.5 g/L KCl, 2 g/L NaNO_3_, 30 g/L sucrose, 20 g/L agar, 200 μl/L of a trace element solution that was added after autoclaving) plates. Conidia of the *Fusarium* strains were generated by inoculating 50 mL of mung bean extract (MBS, filtrate of 10 g mung beans per L water boiled for 20 min) in a 250 mL baffled flask with fungal mycelium. After 3 days of incubation on a shaker at 140 rpm at 20 °C in the dark, conidia were obtained by removing mycelium using sterilized glass wool and subsequent sedimentation overnight at 4 °C. Five hundred spores were spotted onto FMM plates containing different concentrations of FB_1_. *Aspergillus* and *Alternaria* strains were grown on potato dextrose agar (PDA, Sigma-Aldrich, Vienna, Austria). Agar blocks from colonies grown on PDA were transferred onto plates containing different concentrations of FB_1_. The plates were supplemented with different concentrations of a crude FB_1_ extract that was previously used for yeast spottings [34]. Pure fumonisin was purchased from Fermentek (Jerusalem, Israel) and Fumizol Ltd. (Szeged, Hungary), and the 70% pure FB_1_ was a gift from Romer Labs. The plates were incubated at 20 °C and pictures were taken after 5 days.

### 4.2. Generation of Δfum1, Δfum17-18 Mutants

The fumonisin-nonproducing Δ*fum1* mutant GfA2364 containing a hygromycin resistance cassette disrupting the coding region of *FUM1* polyketide synthetase was kindly provided by Dr. Robert Proctor. *FUM1* disruption was confirmed by using primers hyg-FW and hyg-RV to amplify an internal 861 bp hygromycin fragment, as well as by implementing primers flanking the insertion site (primers GfA2364_fum1test_fw and GfA2364_fum1test_rv), leading to a 2.9 kb fragment (Table 2). 

A double knock-out of putative self-protection genes *FUM17* (FVEG_00327) and *FUM18* (FVEG_00328) was performed in strain GfA2364 by replacing them with a geneticin resistance marker, *nptII* (G418). The 5′ UTR upstream of the FVEG_00328 promoter was amplified from *F. verticillioides* genomic DNA using the primers Fw_Fum328KO and Rv_Fum328KO, while the downstream UTR of FVEG_00327 was obtained using the primers Fw_Fum327KO and Rv_Fum327KO. The 5′ UTR was digested with BcuI and EcoRI and ligated into vector pKT300 containing a fusion gene between HSV-thymidine kinase and nptII [50]. Likewise, the 3′ UTR, was also cloned into pKT300 using HindIII and SalI. Finally, they were cloned into the same disruption plasmid, named pKT314. GfA2364 was transformed using a standard transformation protocol [50]. The knockout was confirmed by using the primers FUM1718_downstr_PCRtest, located downstream of *FUM17*, in combination with #940 (inside terminator region of disruption plasmid), located inside the disruption plasmid, as well as FUM1718_upstream_PCRtest, upstream of *FUM18* together with #926 (inside promoter region of disruption plasmid). Both of these amplifications lead to the expected 1 kb fragment, while the control (GfA2364) did not give a band. Primary transformants were obtained and purified to generate homokaryotic transformants. To this end, conidiospores were re-isolated from single colonies for two rounds while maintaining selection pressure to generate second-generation transformants. Primer sequences and purposes are given in Table 2.

### 4.3. Expression of Putative Self-Protection Genes in an FB_1_-Sensitive Baker’s Yeast Strain 

Plasmid pYes2-P_TEF1_ [31] was used to express predicted fumonisin self-protection genes. It contains *URA3* as a selection marker, with genes expressed under the constitutive yeast *TEF1* promoter. Plasmids containing *CER1*, *CER2*, *CER3*, *LAG1*, *LAC1*, *FUM17*, and *FUM18* were described in [31] and kindly provided by Dr. Vito Valiante (Leibniz Institute for Natural Product Research and Infection Biology, Hans Knöll Institute, Jena, Germany). The fumonisin-sensitive baker’s yeast YTKT33 [34] was transformed with these plasmids using the lithium transformation protocol and selected on synthetic complete media lacking uracil (SC-URA). For plate assays, liquid overnight cultures were diluted back to an OD_600nm_ of 0.1. After reaching an OD_600nm_ of about 0.3, they were diluted to an OD_600nm_ of 0.1, 0.01, and 0.001, and 3 µL of these suspensions was spotted on the agar plates. Photographs were taken after a 5-day incubation period at 30 °C.

## Figures and Tables

**Table 1:** Fungal strains used in this study.

Species	Strain Designation (Other Collection)	Genotype
*Fusarium verticillioides*	FGSC 7600; (FRC M-3125, NRRL 20956)	wt ^1^
*Fusarium graminearum*	PH-1 (NRRL 31084)	wt
*Alternaria alternata* f.sp. *lycopersici*	AS27-12	wt
*Alternaria alternata (mali)*	MA 304 (CBS 106.24, ATCC 13963)	wt
*Alternaria alternata*	MA 308 (CBS 150.24)	wt
*Aspergillus niger*	ATCC 11414	wt
*Aspergillus nidulans*	FGSC A4 (ATCC 38163)	wt
*F. verticillioides*	GfA2364	*fum1*::*hygB*
*F. verticillioides*	KTFD1 KTFD4	*fum1*::*hygB fum17-18*Δ::*HSVtk-nptII* (this study)

^1^ wt (wild-type).

**Table 2:** Primers used in this study.

Name	Sequence
Δfum1 confirmation	
GfA2364_fum1test_fw	AGAAGCCTTGATGCTGCCTA
GfA2364_fum1test_rv	GAGTGATGTCCCATGGCAGA
hyg-FW	GCTTTCAGCTTCGATGTAGGAGG
hyg-RV	CTACACAGCCATCGGTCCAGAC
Δfum17,18 disruption	
Fw_Fum327KO	ACTAGTCACGACAGTAAGAAGCAA
Rv_Fum327KO	GACTTGACGGGGATCGGTTC
Fw_Fum328KO	GGATTTGGAGACAAGTACGA
Rv_Fum328KO	GTCGACATCCTTCTCGAAGGCCAG
P#926	TGCTCCAACTCAGGCGATGCTG
P#940	CCGTCTAGCGCTGTTGATTGTATT
FUM1718_upstream_PCRtest	GCCTTCAAAGTTCATCATGGC
FUM1718_downstr_PCRtest	TAAGCGTGTCGTAACCTGTG

## Data Availability

The original contributions presented in the study are included in the article, further inquiries can be directed to the corresponding author.
